# Recognizing Degenerative Aging as a Treatable Medical Condition: Methodology and Policy

**DOI:** 10.14336/AD.2017.0130

**Published:** 2017-10-01

**Authors:** Ilia Stambler

**Affiliations:** Department of Science, Technology and Society, Bar Ilan University, Israel

**Keywords:** senescence, aging, aging-related diseases, frailty, diagnosis, regulation

## Abstract

It is becoming increasingly clear that in order to accomplish healthy longevity for the population, there is an urgent need for the research and development of effective therapies against degenerative aging processes underlying major aging-related diseases, including heart disease, neurodegenerative diseases, type 2 diabetes, cancer, pulmonary obstructive diseases, as well as aging-related complications and susceptibilities of infectious communicable diseases. Yet, an important incentive for the research and development of such therapies appears to be the development of clinically applicable and scientifically grounded definitions and criteria for the multifactorial degenerative aging process (or “senility” using the existing ICD category), underlying those diseases, as well as for the safety and effectiveness of interventions against it. Such generally agreed definitions and criteria are currently absent. The devising of such criteria is important not only for the sake of their scientific value and their utility for the development of therapeutic solutions for the aging population, but also to comply with and implement major existing national and international programmatic and regulatory requirements. Some methodological suggestions and potential pitfalls for the development of such criteria are examined.

Given the rapid aging of the world population and the accompanying rise of aging-related diseases and disabilities, the task of increasing the healthy and productive period of life becomes an urgent global priority. It is becoming increasingly clear that in order to accomplish this purpose, there is an urgent need for effective therapies against degenerative aging processes underlying major aging related diseases, including heart disease, neurodegenerative diseases, type 2 diabetes, cancer, pulmonary obstructive diseases, as well as aging-related complications and susceptibilities of infectious communicable diseases. Arguably, the research, development and distribution of such therapies need to be accelerated [1-4]. But how? One facilitating possibility may be to recognize the degenerative aging process itself as a medical problem to be addressed [5-7]. Such recognition may accelerate research, development and distribution in several aspects: 1) The general public will be encouraged to actively demand and intelligently apply aging-ameliorating, preventive therapies; 2) The pharmaceutical and medical technology industry will be encouraged to develop and bring effective aging-ameliorating therapies and technologies to the market; 3) Health insurance, life insurance and healthcare systems will obtain a new area for reimbursement practices, which will encourage them and their subjects to promote healthy longevity; 4) Regulators and policy makers will be encouraged to prioritize and increase investments of public funds into aging-related research and development; 5) Scientists and students will be encouraged to tackle a scientifically exciting and practically vital problem of aging.

Yet, in order for degenerative aging process to be recognized as a diagnosable and treatable medical condition and therefore an indication for research, development and treatment, a necessary condition appears to be the development of evidence-based diagnostic criteria and definitions for degenerative aging. Such commonly accepted criteria and definitions are currently lacking. Yet without such scientifically grounded and clinically applicable criteria, the discussions about “ameliorating” or even “curing” degenerative aging processes will be mere slogans. Such criteria are explicitly requested by major regulatory frameworks, such as the International Classification of Diseases (ICD), the WHO Global Strategy and Action Plan on Ageing and Health (GSAP), the European Medicines Agency (EMA), the US Food and Drug Administration (FDA). Nonetheless, nobody has yet done the necessary work of devising such criteria. The present article will discuss some methodological suggestions and potential pitfalls for their development.

## Regulatory and policy frameworks

“Senility,” tantamount to degenerative aging, is already a part of the current ICD-10 listing, carrying the code R54. The “senility” code is also applicable to “old age,” “senescence” and “senile asthenia” as well as “senile debility,” while excluding “senile psychosis” (F03) (http://apps.who.int/classifications/icd10/browse/2016/en#R54). In the draft ICD-11 version (to be finalized by 2018, www.who.int/classifications/icd/revision/en/), the code MJ43 refers to “Old age,” synonymous with “senescence” and “senile debility,” while excluding “senile dementia” (code AA60) (http://apps.who.int/classifications/icd11/browse/l-m/en#/http://id.who.int/icd/entity/835503193). The nearly 40 associated “index terms” in the ICD-11 draft also include “ageing” itself, “senility” (n.o.s.), “senile degeneration,” “senile decay,” “frailty of old age” and others. Still, the current definitions, such as “senility” defined in an ICD-11 draft as “failure of function of otherwise normal physiological mental or physical process(es) by aging. Not to be used under the age of 70 years” seem to be rather deficient in terms of their clinical utility. Furthermore, a comprehensive, scientifically and clinically usable list of general symptoms for “senility” in the ICD is still lacking. This may be the reason why “senility” has been commonly considered a “garbage code,” e.g. in the Global Burden of Disease (GBD) studies [8,9]. The reason “senility” has been considered a “garbage code” is likely because there have been no reliable, clinically applicable and scientifically grounded criteria for diagnosis of “senility” or of “senile degeneration.” Consequently, there could be no official case finding lists. Hence, in order to successfully use this code in practice, it appears to be necessary to be able to develop formal and measurable, biomarkers-based and function-based diagnostic criteria for “senility” or “senile degeneration,” as well as measurable agreed means to test the effectiveness of interventions against this condition.

The need to develop diagnostic criteria for degenerative aging, including evidential biomarkers, as well as functional and clinical end points, is also directly suggested by the recent World Health Organization’s “Global Strategy and Action Plan on Ageing and Health (GSAP) - 2016-2020” (November 2015, http://www.who.int/ageing/global-strategy/en/). The GSAP includes “Strategic objective 5: Improving measurement, monitoring and research on Healthy Ageing,” with a clause “5.1: Agree on ways to measure, analyse, describe and monitor Healthy Ageing” (Section 95). It acknowledges that “The current metrics and methods used in the field of ageing are limited, preventing a comprehensive understanding of the health issues experienced by older people and the usefulness of interventions to address them. Consensus should be reached on common terminology and on which metrics, biological or other markers, data collection measures and reporting approaches are most appropriate.” Thus, WHO clearly and explicitly articulated the need to devise scientifically grounded and clinically applicable definitions, criteria and measures for aging, and for clinical interventions into it. These statements by the WHO GSAP imply support for biomedical diagnostic and therapeutic research of aging.

Some international policy documents and programs are less supportive of such research, still some support may be derived from them. For example, within the UN “Sustainable Development Goals (SDG) - until 2030” (adopted in September 2015, https://sustainabledevelopment.un.org/sdg3), the Sustainable Development Goal - SDG 3 “Ensure healthy lives and promote well-being for all at all ages” mandates: “By 2030, reduce by one third *premature* mortality from non-communicable diseases through prevention and treatment” (3.4., emphasis added). This implies that “mature” mortality is somehow acceptable, as well as suggests the need to provide diagnostic criteria for the discrimination of “premature” mortality. This clause omits or does not explicitly mention the aged and the processes of aging (the formulation “for all ages” itself makes the aging problem rather inconspicuous, not prioritized). Yet it may be argued that it is only by prevention and treatment of the underlying aging processes, thanks to biomedical research and development, that the goal of a significant reduction of mortality from non-communicable age-related diseases could ever be achieved. The SDG3 Clause 3.b mandates that the global community should “Support the research and development of vaccines and medicines for the communicable and non-communicable diseases that primarily affect developing countries, provide access to affordable essential medicines and vaccines.” Apparently, this statement undervalues the support for research of aging-related diseases that presumably primarily affect the “developed” (a.k.a. “high income”) countries, thus implying both that the aging plagues of the developed countries are not a research priority and that those plagues are irrelevant for the “developing” (“low income”) countries. Yet, in fact, aging-related morbidity is an ever-increasing concern for the developing countries, while their gerontological and geriatric infrastructure is far less advanced than in the developed countries [10]. The introduction of enhanced science-based medical evaluation criteria for the aged may contribute to the development of gerontological research and practice capabilities in the developing countries, also as a part of the SDG framework.

At the national level, for over two decades, the regulatory authorities of the EU, US and Japan have struggled to obtain special consideration for older patients in the research, development and application of medical treatments, to involve elderly subjects in all clinical trials, and to establish criteria for treatment efficacy and safety specifically for the elderly. Thus, in 1993, “The International Conference on Harmonisation of Technical Requirements for Registration of Pharmaceuticals for Human Use (ICH)” issued the “Harmonized Tripartite Guideline E7” (recommended for adoption in the EU, US and Japan) regarding “Studies in Support of Special Populations: Geriatrics” (http://www.ich.org/). This guideline posited the general principle that “Drugs should be studied in all age groups, including the elderly, for which they will have significant utility.” Still, this basic requirement has not yet become an overwhelming practice, while comprehensive criteria for the special medication needs of older patients, in particular the efficacy and safety criteria for the elderly, are still deficient or even lacking in many studies.

In the EU, in the past years, the European Medicines Agency (EMA) has undertaken several programmatic initiatives to include the elderly into clinical trials and to develop the relevant diagnostic, inclusion, efficacy and safety criteria (http://www.ema.europa.eu/ema/). Thus, in February 2011, the EMA issued the “EMA geriatric medicines strategy” that would “ensure that the needs of older people are taken into account in the development and evaluation of new medicines” [11]. Yet, subsequent reports revealed that those needs, in many cases, are not sufficiently addressed [12]. There have been also continuous efforts by the EMA to develop the diagnostic criteria for general age-related frailty as a common determinant of age-related diseases and disabilities. Thus, in March 2013, the EMA issued the brief “Concept paper on the need for a reflection paper on quality aspects of medicines for older people.” The paper urged to reflect on the fact that “there is no specific legal requirement for the development of medicines for geriatric use” [13]. Also about the same time, in May 2013, the EMA issued the “Proposal for the development of a points to consider for baseline characterisation of frailty status” including physical frailty, comorbidity status and mental frailty [14]. Apparently, these documents are still in preparation [15].

The situation at the US Food and Drug Administration (FDA) appears to be similar. The need for the inclusion of older subjects in all clinical trials and the necessity for devising specific criteria for their diagnostic and therapeutic assessment are recognized. Thus, following the “ICH guidance E7 Studies in Support of Special Populations: Geriatrics,” in 2012, similarly to the EMA, also the FDA expressed the hope that “certain specific adverse events and age-related efficacy endpoints should be actively sought in the geriatric population, e.g., effects on cognitive function, balance and falls, urinary incontinence or retention, weight loss, and sarcopenia” (www.fda.gov/downloads/drugs/guidancecomplianceregulatoryinformation/guidances/ucm189544.pdf). Yet, apparently, this directive has not been satisfactorily accomplished. For example, there is no mandatory inclusion of elderly subjects in NIH trials, unlike children, women and minorities (https://humansubjects.nih.gov/).

Nonetheless, an important development recently occurred with the FDA. In November 2015, the FDA approved the “TAME” study - “Targeting Aging with Metformin,” which should evaluate the ability of metformin (a well known anti-diabetic, anti-glycemic medication) to reduce or postpone multiple age-related diseases and dysfunctions [16]. The study, perhaps for the first time, is approved for an intervention into the aging process in order to reduce aging-related multimorbidity. Indeed, the study does not measure the effects on *“aging”* as such (for which there is currently no agreed measurable clinical definition or criteria), but on multiple age-related diseases and dysfunctions (which can be clinically diagnosed and which together are termed “multimorbidity” or “comorbidity”). Yet, crucially, there is no agreed measurable clinical definition and criteria for multimorbidity either [17,18]. The developments of agreed and strict methodologies to evaluate either degenerative aging itself or age-related multimorbidity or frailty, as treatable medical conditions, still appear to be desirable tasks for the future, for the EMA, FDA and other national regulatory agencies.

## Frailty evaluation

Notably, “treatable medical condition” does not necessarily mean a “disease.” “Medical condition” in relation to aging may as well refer to “geriatric syndrome” as is commonly used by geriatricians to define aging-related frailty (indicative of “high risk for a number of adverse health outcomes, and mortality” [19]), as well as delirium, falls, and incontinence. However, in order to arrive at reliable diagnostic definitions, the common functional frailty assessments may need to be supplemented in a larger scope with assessments of biomarkers of the aging process, in correlation with each other, thus reinforcing the diagnostic and predictive capacity of the combined functional and biological indicators. Currently, functional assessments dominate the evaluations of frailty (http://frailty.net/diagnostic-tools/). For example, in the widely used “Study of Osteoporotic Fractures” (SOF) frailty index, there are 3 main diagnostic parameters: 1) “*Weight loss,” 2) “Inability to rise from a chair,”* and *3) “Poor energy”* as identified by an answer “yes” or “no” to the question “Do you feel full of energy?” on the Geriatric Depression Scale [20]. And in the even more widely used “Cardiovascular Health Study” (CHS) frailty index, the 5 parameters are: 1) “*Shrinking”* as shown by an unintentional weight loss, 2) “*Weakness”* as shown by a maximal grip strength, 3) “*Poor energy”* as determined by an answer to the question “Do you feel full of energy?” 4) “*Slowness”* as indicated by an average walk speed, and 5) “*Low physical activity level*” as identified by a Physical Activity Scale for the Elderly (PASE) score in the lowest quintile [21]. It may be seen that biological markers of aging are assigned little significance in such scores.

Yet, it has been suggested to use frailty assessments, in particular the “accumulation of deficits” in frailty, as a “proxy measure of the aging process” [22]. However, in order to provide a reliable science-based proxy or indication for the aging process, it appears necessary to include more parameters measuring this process at its fundamental biological level. For example, the organism’s energy level can be objectively measured by such means as spirometry, oximetry, hemodynamic, electrochemical and spectroscopic energy metabolite measurements, etc., thus providing improved indication for therapy [23,24]. The energy metabolism measurements may supplement molecular-biological measurements that are commonly employed in the research of biomarkers of aging (e.g. age-related changes in telomere length, advanced glycation endproducts - AGE, DNA repair capacity, aging-associated gene expression and epigenetic markers, stem cell populations and others) [25].

The addition of biological indicators to frailty assessments may provide advanced diagnostic capabilities. Yet, in many cases, age-related frailty is not considered among the clinical assessments of the aged at all, even based on only functional parameters. In policy, one way to emphasize the biological and therefore the medically treatable components of the “geriatric syndrome” or “frailty” may be through the WHO’s “International Classification of Functioning, Disability and Health (ICF)” (http://www.who.int/classifications/icf/en/). It appears to exist in parallel, and apparently not strongly related to either ICD or GSAP, and hardly even mentions aging as such or the “intrinsic capacity” in aging which is the focus of the GSAP. The addition of biomedical tests on aging to the ICF may be parallel to an addition of some clinically applicable, science-based and practical definitions, criteria or classification of aging or senility within the ICD and GSAP. It may be possible to emphasize the clinical significance of the aging process on all the fronts at once.

## Methodological challenges

This work has argued that the devising of clinical criteria for degenerative aging or “senility” (to use the current ICD term) is important not just for the sake of their scientific interest, or their utility for developing therapeutic solutions for the aging population, but also to comply with and implement major national and international programmatic and regulatory requirements. Yet several critical methodological challenges may arise in developing commonly acceptable diagnostic definitions and criteria for degenerative aging.

A major challenge is related even to the semantic understanding of the term “degenerative aging.” The term “degenerative” may imply both the present state of degeneration and the process leading to the state of degeneration. This distinction may have major implications for intervention, respectively implying a curative approach to the already manifest state of degeneration (a late stage intervention) as opposed to a preventive approach to block a process leading to degeneration (an early stage intervention). Here the term “degenerative aging” is understood mainly in the latter sense, as a process leading to degeneration that can be prevented.

Yet, many questions remain with such a definition. Obviously, not every time-related change leads to degeneration and disease, and some aging-related changes may be beneficial for the person (e.g. the proverbial “wisdom of age” [26]). Obviously also, many changes leading to age-related degeneration begin at conception, and may be necessary concomitants of the processes of growth and development. Then for which processes and at which stages is intervention warranted? In other words, which aging processes can be considered truly “degenerative” (leading to degeneration) that would require preventive intervention? Several sets of such candidate processes have been proposed [27-29], yet there is still little empirical evidence that intervention into them will have clinical benefits. The potential interrelation and regulation of these various processes are also uncertain. In this regard, a practical worry is that under the title of “prevention” and “early intervention” - drugs and other treatments will be sold to young and relatively healthy individuals without a real need and without proven benefits in actually preventing degenerative states. A more thorough, quantitative and formal understanding of old-age degeneration (frailty) as a physiological state is required as well. Should it be measured as a lack of function and adaptation to the environment, an impairment of homeostatic or homeodynamic stability? [17,30] Should it be presented as an index or as physiological age [31]?

Each of these options would raise a host of questions of its own, whose mere mentioning would go far beyond the scope of this policy review. To provide evidence-based answers to those questions, vast empirical and theoretical research yet appears to be needed to establish diverse age-related changes as predictors of adverse age-related outcomes (such as multi-morbidity and mortality) as well as evaluate the effects of various preventive and curative treatments on those outcomes. Based on such data, better formal, clinically applicable models of degenerative aging as a process and as a state can be developed. It is hoped that the present work will contribute to raising the demand for more such research.

Just a few particular challenges may be mentioned here for the development of diagnostic and treatment criteria for degenerative aging. These can be tentatively classified as follows: 1) establishing definitions, 2) minimizing confounding factors, 3) improving informative value, and finally 4) improving the practical utility of the criteria. This could also be the putative priority order at which the problems can be tackled. (It must be reemphasized that these propositions are only intended to stimulate academic and policy discussion.)

### I. Establishing definitions

#### 1) Establishing basic terms and definitions

These may include the questions above. For example, should “degenerative aging” be understood as a process or as a state? Or is “healthy aging” a helpful term for developing clinical measurements of aging, considering that most aging processes increase morbidity? Should we instead speak in terms of “healthy longevity” as opposed to “degenerative aging”?

#### 2) Defining clinical benefits

Just and only biomarkers of aging may not be sufficient to provide clinically applicable diagnostic criteria for “degenerative aging” or for interventions against it. There is a need to precisely define measurable *clinical* end points, demonstrating evidential clinical benefits [32,33], especially for the reduction of age-related multimorbidity. The combination of structural biological and functional behavioral parameters may increase diagnostic capabilities.

### II. Minimizing confounding factors

#### 1) Focus on older persons

The clinical benefits need to be evaluated in the primary target population - the older frail persons, rather than the younger and healthier ones who may exhibit entirely different biological responses [34].

#### 2) Long term consideration

The clinical criteria and biomarkers, as well as resources available to the organism, need to be considered *for the long term* [35]. Thanks to long-term evaluation it may be possible to control for effects of over-stimulation, as well as rule out transient compensatory and psychosomatic effects and seeming short-term benefits that may arrive at the expense of long-term deterioration. In particular, seeming short-term “rejuvenation effects” may increase mortality and shorten the actual lifespan [36].

### III. Improving informative value

#### 1) Selection

As almost any age-related biological parameter may be considered a “biomarker of aging,” there is a need to select the most predictive and economic biomarkers, for the population as well as for individuals [37].

#### 2) Integration

Criteria for degenerative aging may not be only molecular and cellular, but at every level of biological organization - from the molecular to cellular to tissues and organs, to the entire organism and to the organism’s interrelation with the environment - that need to be integrated [38]. Moreover, these criteria may not necessarily be chemical and biological, but can also be physical, in particular as relates to various resuscitation technologies as applied to the elderly, such as hypothermia and suspended animation [39], oxygenation and energy metabolism [40], electromagnetic stimulation [41]. Social (engagement) and psychological (motivation) criteria also need to be added.

#### 3) Interrelation and balance

Individual biomarkers may not be indicative of the process or state of degeneration, and need to be considered in combinations, or ideally in a systemic balanced way - otherwise interventions on particular biomarkers and pathways may exacerbate other biomarkers and pathways, and disrupt the system as a whole. The general methodology for the evaluation of the effects of multiple risk factors (including biomarkers of aging) on multiple age-related diseases (multimorbidity) need to be improved, to allow the evaluation of non-linear, cumulative or synergistic effects [31,42,43].

### IV. Improving practical utility

#### 1) Pluralism and rigor

Particular batteries of assays are usually related (and potentially biased) to particular theories, research agendas, academic schools and commercial interests. There is an apparent need to allow pluralism of investigation, discovery and application, while maintaining standards of the scientific method. Consensus standards often emerge as a result of data-sharing [44], which may become a practical challenge of its own.

#### 2) Affordability

Costs of biomarkers assays may become prohibitive or even impractical for use by most people in the world. There is a need to focus on such biomarkers and functional assays that may be most cost-effective, especially those that are already routinely used in clinical practice, while still encouraging the development of more sophisticated assays, that may become more accessible in time, and specifically devising means to increase their accessibility [45].

All these issues must become a subject of massive and pluralistic consultation, involving scientists, policy makers and other stakeholders. Thanks to such a consultation it may be possible to develop agreeable scientific clinical criteria for degenerative aging that could improve diagnostic capabilities and allow better informed clinical decisions, as well as stimulate further research and development of effective, evidence-based therapies treating the underlying processes of aging-related diseases rather than their particular symptoms.
